# Uniting the neurodevelopmental and immunological hypotheses: Neuregulin 1 receptor ErbB and Toll-like receptor activation in first-episode schizophrenia

**DOI:** 10.1038/s41598-017-03736-3

**Published:** 2017-06-23

**Authors:** Szabolcs Kéri, Csilla Szabó, Oguz Kelemen

**Affiliations:** 1 Nyírő Gyula Hospital – National Institute of Psychiatry and Addictions, Budapest, Hungary; 2 Department of Cognitive Science, Budapest University of Technology and Economics, Budapest, Hungary; 3 Department of Physiology, Faculty of Medicine, University of Szeged, Szeged, Hungary; 4 Department of Behavioral Sciences, Faculty of Medicine, University of Szeged, Szeged, Hungary

## Abstract

Current pathophysiological models of schizophrenia focus on neurodevelopmental and immunological mechanisms. We investigated a molecular pathway traditionally linked to the neurodevelopmental hypothesis (neuregulin 1 - ErbB), and pathogen-associated pattern recognition receptors associated with the immune hypothesis (Toll-like receptors, TLRs). We recruited 42 first-episode, drug-naïve patients with schizophrenia and 42 matched healthy control subjects. In monocytes TLR4/TLR5 and ErbB expressions were measured with flow-cytometry. Pro-inflammatory cytokines (IL-1β, IL-6, and TNF-α) and the anti-inflammatory cytokine IL-10 were determined following the stimulation of TLR4/TLR5 and ErbB. Results revealed increased TLR4/TLR5 and decreased ErbB4 expression in schizophrenia relative to the control subjects. The expression of ErbB2 and ErbB3 receptors was unaltered in schizophrenia. TLR4 stimulation resulted in lower pro-inflammatory cytokine production in schizophrenia compared to the control levels, whereas the stimulation of ErbB by neuregulin 1 led to higher pro-inflammatory cytokine levels in patients with schizophrenia relative to the control group. In healthy controls, ErbB activation was associated with a marked production of IL-10, which was dampened in schizophrenia. These results indicate that the stimulation of TLR4 and ErbB induces opposite pro-inflammatory cytokine responses in schizophrenia.

## Introduction

The etiology and pathophysiology of schizophrenia are one of the most multifaceted and controversial areas of research into neuropsychiatric disorders. Beyond the classic neurodevelopmental hypothesis, supposing an interaction between inherited genetic factors and environmental insults^1–3^, there is a renewed and emerging interest regarding the putatively underlying immunological alterations in schizophrenia^4–6^. However, the neurodevelopmental and immunological hypotheses are not mutually exclusive^7^. There is evidence that immunological and inflammatory mechanisms play a substantial role in early and late neurodevelopmental processes, as well as in the regulation of neurotransmission and synaptic plasticity^8–10^.

Several “historical candidate genes” of schizophrenia discovered in the past decades have been shown to be related to neurodevelopment and synaptic plasticity, but, disappointingly, new generations of genome-wide associations studies failed to replicate most of these findings^11,12^. New molecular genetic data, based on genome-wide association studies with better statistical power, suggest that major histocompatibility complex class I (MHC-I) molecules may form a bridge between genetic predisposition, immunological mechanisms, and environmental risk factors (e.g., maternal infection, obstetric complication, and stress) by regulating neurite growth, synapse formation, and activity-dependent synaptic tuning in the brain^13^. Another new candidate is the complement system, a complex network of more than 30 plasma proteins facilitating antigen-antibody interactions and coating pathogens, which are also implicated in synaptic pruning in the brain^14,15^.

A somewhat neglected observation is that the products of some “historical candidate genes” also have an impact on immune functions. A notable example is the profoundly characterized molecule neuregulin 1 (NRG1) and its ErbB receptors (ErbB4 and ErbB2/ErbB3 complex)^16–21^. A timely and detailed review and a meta-analysis by Mostaid *et al*.^16,22^ demonstrated that, despite the fact that whole-genome association studies did not confirm a link between NRG1/ErbB genes and schizophrenia, there is substantial evidence from other types of preclinical and clinical studies that NRG1 may be relevant in the pathophysiology of schizophrenia by regulating not only synapse formation but the balance between excitatory (glutamatergic) and inhibitory (GABA-ergic) neurotransmission.

In addition to these neuronal effects, NRG1 can modulate the functions of peripheral immune cells. Sei *et al*.^23^ demonstrated that NRG1-induced cell migration of B lymphoblasts is decreased in schizophrenia, which is at least partly mediated by impaired AKT signaling stimulated by the tyrosine-kinase receptor ErbB4. The authors hypothesized that this phenomenon might be a peripheral immunological marker of central nervous system developmental anomalies^23^. ErbB3, but not ErbB2, mRNA levels were significantly reduced in the lymphoblasts of schizophrenia patients, and it was not associated with the ErbB4 risk haplotype^24^. We showed that impaired NRG1-induced B cell activation correlates with weak physiological habituation of arousal and paranoid ideas^25^, abnormal gating of perceptual information^26^, and decreased hippocampal volume in schizophrenia^27^. Furthermore, additional evidence from animal and human studies suggests that NRG1 may be a key factor in the interplay of neurodevelopment, synaptic plasticity, glutamatergic/GABA-ergic neurotransmission, and immunological mechanisms in schizophrenia^28–35^.

Recently, Crisafulli *et al*.^36^ conducted a thorough molecular network analysis and found that 60 single-nucleotide polymorphisms of 30 schizophrenia-associated genes are related to pathways of neurodevelopment, apoptosis, vesicle traffic, immune response, and the mitogen-activated protein kinase (MAPK) pathway, a characteristic pattern of Toll-like receptor (TLR) cascade. TLRs are implicated in the first-line detection of pathogens and danger-related signals indicating tissue damage^37–40^. However, the functional relationship between NRG1/ErbB and TLRs has not been elucidated.

TLRs can be found on the membrane of macrophages and other immune cells (TLR1, TLR2, TLR4, TLR5, TLR6, and TLR11 are inserted into cell membranes; TLR3, TLR7, TLR8, and TLR9 are localized to intracellular compartments). The first element of the TLR signaling pathway is the cytoplasmic Toll/Interleukin-1 (IL-1) receptor (TIR) domain, which is linked to an adaptor, MyD88^37–40^. An analysis of the statistical epistatic network of NRG1 in clinical studies of schizophrenia indicated its relationship with IL-1β^16^, which may be an important link to TLR signaling.

TLRs do not exclusively participate in peripheral immunological functions. For example, TLR3 and TLR4 have an impact on neurodevelopment, neuronal survival, and plasticity by regulating the interactions between the immune system and neurons in the developing brain^10,41^. Consistent with this function, several studies have demonstrated an altered expression and activation of TLRs in schizophrenia in immune cells and neuronal elements^42–46^. Recently, we demonstrated up-regulated TLR4 and TLR5 on monocytes and lymphocytes of drug-naïve schizophrenia patients; TLR4, but not TLR5, alterations were normalized by antipsychotic treatment^47^. In the drug-naïve state, a higher expression of TLR4 was associated with worse cognitive functions, indicating that abnormal peripheral TLR expression may be linked to central nervous system processes in schizophrenia^47^.

The results discussed above raise the possibility that the NRG1/ErbB and the TLR systems may interact in schizophrenia. However, this hypothesis has not been investigated empirically. Therefore, in the present study, we specifically focused on the expression and activation of TLRs and ErbB on the monocytes of 42 drug-naïve, first-episode schizophrenia patients. A considerable amount of data from previous studies suggest that TLR4 may be especially important in the pathogenesis of schizophrenia and other neuropsychiatric conditions^46^, and monocytes are dominantly affected in schizophrenia^44,47^. TLR5 served as a control to test the specificity of TLR4-related changes.

In addition to receptor expression, we investigated the production of proinflammatory (IL-1β, IL-6, and TNF-α) and anti-inflammatory (IL-10) cytokines by monocytes after stimulation with TLR4/TLR5 ligands (bacterial lipopolysaccharides (LPS) and flagellin, respectively) and NRG1. We hypothesized an abnormal expression and activation of TLRs and ErbB in patients with schizophrenia relative to the control subjects^44,47,48^.

## Results

### Expression of TLRs and ErbB

**Figure 1:**
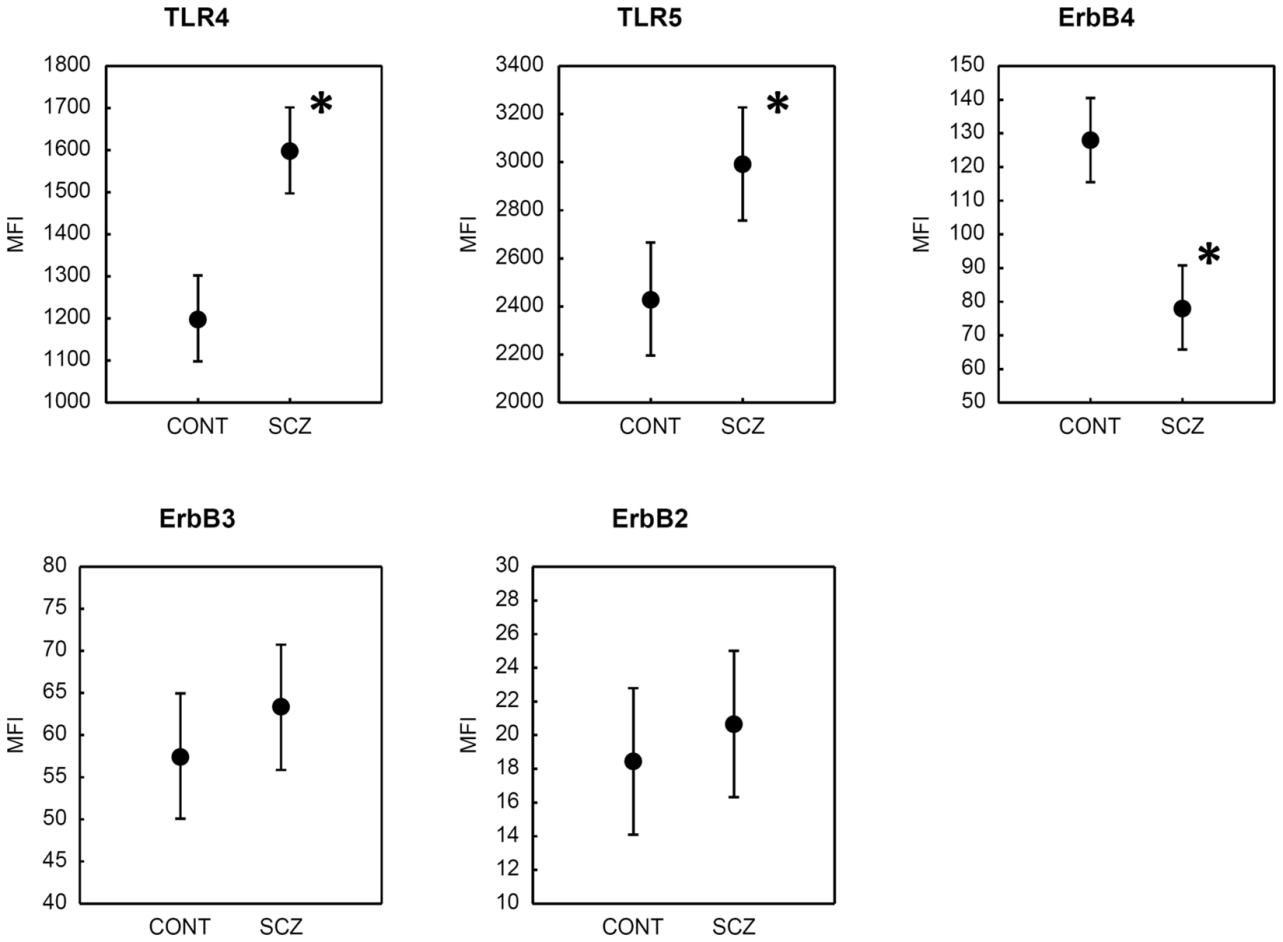
Mean fluorescent intensity (MFI) values for TLRs and ErbB in patients with schizophrenia (SCZ, n = 42) and healthy control subjects (CONT, n = 42) as measured in monocytes by flow cytometry. Error bars indicate 95% confidence intervals. *Significant between-group differences, Tukey HSD, p < 0.01.

There was a significant difference between patients with schizophrenia and healthy control participants in the expression (mean fluorescent intensity (MFI) as measured by flow cytometry) of TLRs and ErbB4: we observed an increased expression of TLR4 (F(1,82) = 30.21, p < 0.001; η^2^ = 0.27) and TLR5 (F(1,82) = 11.27, p < 0.005; η^2^ = 0.12) in patents with schizophrenia relative to the control subjects, whereas in the case of ErbB4, the opposite effect was found (decreased expression in schizophrenia compared to the controls) (F(1,82) = 31.30, p < 0.001; η^2^ = 0.28). There was no significant between-group difference for ErbB2 and ErbB3 (Fig. 1).

### Cytokine production following TLR and ErbB4 stimulation

There was a significant difference between patients with schizophrenia and healthy controls in cytokine production (main effect of group in the ANOVA: F(1,82) = 4.35, p < 0.05; η^2^ = 0.05). However, the difference between patients and controls was modulated by the type of receptor stimulation (TLR4/TLR5 and ErbB ligands) (two-way interaction between group and stimulation type in the ANOVA: F(2,164) = 33.34, p < 0.001; η^2^ = 0.29). Critically, the difference between patients and controls was modulated by the interaction of stimulation type and different cytokines (IL-1β, IL-6, TNF-α, and IL-10) (three-way interaction between group, stimulation type, and cytokines in the ANOVA: F(6,492) = 11.55, p < 0.001; η^2^ = 0.12).

**Figure 2:**
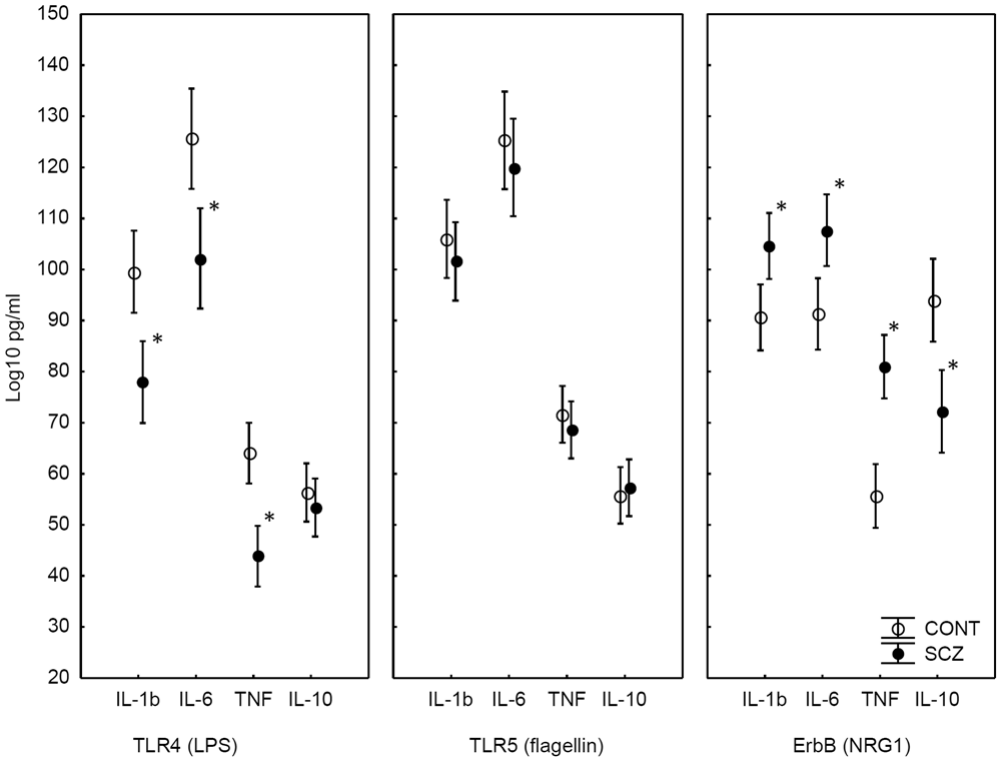
Mean concentration of cytokines following TLR and ErbB4 stimulation in patients with schizophrenia (SCZ, n = 42) and healthy control subjects (CONT, n = 42). Error bars indicate 95% confidence intervals. *Significant between-group differences, Tukey HSD, p < 0.05.

The three-way interaction among experimental group, stimulation type, and cytokines was further analyzed with Tukey HSD tests. In the case of TLR4 stimulation, there were significantly weaker IL-1β, IL-6, and TNF-α responses in schizophrenia relative to the control subjects (p < 0.01), whereas IL-10 production was intact (p > 0.5). In the case of TLR5 stimulations, there were no significant differences between patients and controls (p > 0.5) (Fig. 2).

Finally, in the case of ErbB stimulation by NRG1, we observed a very different pattern of cytokine secretion. For IL-1β, IL-6 and TNF-α, we observed a significantly higher response in schizophrenia patients than in control subjects (p < 0.05). In contrast, IL-10 response was significantly dampened in schizophrenia as compared to the control values (p < 0.01) (Fig. 2).

### Potential confounding factors and clinical symptoms

We performed a separate series of covariance analyses (ANCOVAs) in which smoking, blood cortisol levels reflecting general stress response, body mass index, waist-to-hip ratio, the duration of untreated psychosis, and the PANSS scores were included in the above described ANOVAs as co-variants. The results remained the same, suggesting that these variables do not account for the between-group differences. Similarly, there was no effect of age and sex (p > 0.2).

## Discussion

The findings of the present study indicate that the activation of monocytes of schizophrenia patients is receptor-dependent. In accordance with previous studies^44,47^, TLR4 displayed an increased expression, and the stimulation of these receptors resulted in a lower production of pro-inflammatory cytokines as compared to that observed in healthy control participants. The key and novel finding of the study was that ErbB receptors were characterized by the opposite pattern of expression and activation: there was a lower expression of ErbB4 in schizophrenia relative to controls, but NRG1-induced activation of ErbB receptors led to an enhanced pro-inflammatory cytokine response. Interestingly, the activation of ErbB resulted in a higher production of the anti-inflammatory cytokine IL-10 in healthy individuals as compared to TLR-related IL-10 responses, and this ErbB-dependent IL-10 release was much less dominant in schizophrenia. This suggests a weakened NRG1-dependent anti-inflammatory response in schizophrenia.

Although we did not find an altered expression of ErbB2/ErbB3 receptors in schizophrenia, it cannot be excluded that the observed cytokine profile following NRG1 stimulation is at least partially due to the abnormal activation of ErbB2/ErbB3 receptors, and not only ErbB4 with reduced expression is responsible for the effect. Indeed, recent evidence indicates that ErbB3 plays an important role in the modulation of monocyte responses^49^. The role of different ErbB receptors must be clarified by further studies. Similarly, a key issue is how TLR and ErbB activation might modulate cytokine production in the brain.

Several previous studies found an increased expression of some splicing variants of ErbB4 in the dorsolateral prefrontal cortex of schizophrenia patients^21,50–52^, as well as in peripheral lymphoblastoid cells^24^. This is in contrast with our present findings indicating a decreased expression of ErbB4 in monocytes. Different methods (measuring receptor protein expression by flow cytometry in this study vs. mRNA splice variants in other studies), distinct cell lines (monocytes in this study vs. neurons from postmortem brains and lymphoblastoid cells in other studies), medication status, and illness duration might account for the differences. In addition, ErbB4 expression is influenced by schizophrenia risk haplotypes, and individuals homozygous for the non-risk haplotype exhibit undetectable levels of ErbB4 in lymphoblastoid cells^24^. We did not evaluate ErbB4 haplotypes in our participants.

By using LPS and polyI:C stimulation, Muller *et al*.^44^ found higher TLR4 and TLR3, but not TLR2, expression in monocytes of patients with schizophrenia, which was replicated and extended in an independent sample of drug-naïve patients^47^. Muller *et al*.^44^ also demonstrated a weakened intracellular IL-1β response in the same patients, which suggests a blunted function and a compensatory up-regulation of TLRs. Monocyte hypofunction may lead to a reduced clearance of pathogens and a consequent low-grade inflammation^5,53,54^, which may have a negative impact on cognition and cardiovascular health of the patients. The present data suggest that blunted monocyte function is not a general phenomenon: in the case of ErbB4, we observed an enhanced response together with a down-regulation of the receptor. Moreover, altered monocyte activation was confined to a specific pattern recognition receptor, namely, TLR4. Although TLR5 was also up-regulated in schizophrenia, the stimulation of this receptor resulted in a pro-inflammatory cytokine response comparable to that measured in control subjects. The dissociation between TLR4 and TLR5 response might be attributed to their partially distinct intracellular pathways and compartmentalization: TLR4 is linked to TRIF (TIR-domain-containing adapter-inducing interferon-β)/TRAM (translocating chain-associated membrane) protein (in addition to the classic MyD88 pathway), whereas TLR5 activates IRAK4 via MyD88^55–57^. This hypothesis should be tested by future studies.

The convergence point of TLR and ErbB signaling may be nuclear factor κB (NF-κB), which regulates the expression of genes encoding pro-inflammatory proteins (cyclooxygenase 2, inducible nitric oxide synthase, and cytokines such as IL-1β, IL-6, and TNF-α)^58^. However, the effect of NRG1 is thought to be anti-inflammatory. In cultured microglia and macrophages, Mencel *et al*.^59^ demonstrated that treatment with NRG1 produced a 33% decrease in TNF-α levels and an 88% decrease in IL-6 levels. NRG1 may have an anti-inflammatory and neuroprotective function via the up-regulation of α7 nicotinic acetylcholine receptor, a key component not only in neurotransmission and schizophrenia, but also in cholinergic anti-inflammatory responses^60^. Tynyakov-Samra *et al*.^61^ found reduced levels of ErbB4 in monocytes and lymphocytes of patients with multiple sclerosis, characterized by neuro-inflammation and oligodendroglia dysfunction. The reduced production of IL-10 following ErbB stimulation in schizophrenia suggests an enhanced inflammatory phenotype and a dysfunctional anti-inflammatory effect of NRG1^62^.

The present study is not without limitations. First, cytokine levels were measured only after TLR/ErbB stimulation. However, we observed different cytokine profiles following TLR and ErbB stimulation, which is against the possibility that our findings reflect baseline characteristics and that these immunological changes would be apparent without stimulation. Second, we did not investigate neuregulin 1-beta, which is a predominant form in the brain^16^. Third, it has not been explored how antipsychotics may modulate TLR/ErbB expression and activation. Regarding TLR4/TLR5, it has been demonstrated that antipsychotics reduce receptor expression^47^. We focused our current investigations on TLR4/TLR5 because in a previous study we did not find altered expressions of other TLRs^47^.

The alterations of TLR4 is not specific for schizophrenia. Rather, it might be a common feature of stress-related and developmental neuropsychiatric disorders, including schizophrenia, autism, major depressive disorder, and bipolar disorder^45,46,63–65^. The exact mechanism of TLR4 activation in these disorders is not known. A testable hypothesis focuses on stress-related increases in intestinal permeability (“leaky gut”), which results in the translocation of enteral bacteria to the submucosa where they consequently activate TLRs^66–68^.

Law *et al*.^24^ proposed that the NRG1-ErbB4 signaling system may be a potential therapeutic target for schizophrenia with a special reference to phosphoinositide 3-kinase-p110δ inhibitors. Similar suggestions have been raised in relation to TLR4^45^. Our data indicate that a combined approach targeting both ErbB4 and TLR4 receptors may have a higher chance to achieve clinically significant effectiveness. However, there are several open questions. First, future research should delineate how TLRs and ErbB interact in the brain. Another important question is the potential link between peripheral immunological alterations and the clinical course of schizophrenia. The clarification of these issues might markedly improve our chance to uncover new biomarkers and therapeutic targets for schizophrenia.

## Methods

### Participants and clinical assessment

Forty-two drug-naïve patients with first-episode schizophrenia and 42 healthy control subjects matched for age, gender, and education participated in the study at the National Institute of Psychiatry and Addictions, Budapest, Hungary (Table 1). The mean duration of untreated psychosis was 7.6 months (SD = 4.0). For clinical assessment, we used the Structured Clinical Interview for DSM-IV Axis I Disorders, Clinician Version (SCID-CV)^69^, and the Positive and Negative Syndrome Scale (PANSS)^70^ (Table 1). Overall functioning was assessed with the Global Assessment of Functioning (GAF) scale^69^. The exclusion criteria were history of neurological disorders, head injury, inflammatory diseases or current infections, and psychoactive substance misuse. The patients and controls did not receive anti-inflammatory medications at least for 3 months before testing.

**Table 1:** Demographic and clinical characteristics of the participants.

	Schizophrenia (n = 42)	Control subjects (n = 42)
Male/female	29/13	29/13
Age (years)	26.1 (6.8)	26.2 (5.9)
Education (years)	11.0 (2.7)	10.9 (2.5)
Body mass index (BMI)	23.2 (4.1)	22.7 (4.3)
Waist-to-hip ratio	0.79 (0.12)	t1: 0.79 (0.13)
Smokers/non-smokers	22/20	22/20
**Positive and Negative Syndrome Scale (PANSS)**		
Positive symptoms	19.4 (7.9)	
Negative symptoms	13.5 (7.0)	
General symptoms	56.3 (17.1)	

Data are mean (standard deviations) except for male/female and smokers/non-smokers ratios. There were no significant differences between schizophrenia patients and control subjects as revealed by t-tests (for means/SD) and chi-square tests (for distributions) (p > 0.2).

The study was done in accordance with the Declaration of Helsinki. All participants gave written informed consent and the study was approved by the appropriate ethics board (Egészségügyi Tudományos Tanács – TUKEB).

### Cell isolation and flow cytometry

First, we determined leukocyte counts (LH750 instrument, BeckmanCoulter, Hialeah, FL, USA). There were normal leukocyte ranges in patients with schizophrenia and control individuals (cells × 109/L; neutrophils: 1.6–8.1, monocytes: 0.1–1.1, lymphocytes: 0.9–4.9), and there was no significant difference between the two groups (p > 0.3).

The technical details of the protocol were described previously^47,64^. We obtained peripheral blood (20 mL) from the cubital vein (EDTA tubes). Mononuclear cells were isolated by density gradient centrifugation (900 × *g*, 30-min). We used standard culture medium (RPMI-1640, 0.5% gentamicin, 1% glutamine, 1% HEPES, 0.1% fungizone, 10% fetal calf serum; all components from Sigma-Aldrich, Austria). Cells were first stained with anti-CD14 PE/FITC (BD Biosciences) to isolate monocytes. We used anti-TLR4 biotin (BD Biosciences), goat anti-human TLR5 (Santa Cruz Technologies), TLR4 streptavidin FITC (BD Biosciences), and TLR5 FITC donkey anti-goat IgG (e-Biosciences). For the staining of ErbB4, we used mouse and human ErbB4 antibodies (Santa Cruz Technologies) and allophycocyanin-conjugated F(ab)2 against human Fc (Jackson ImmunoReasearch) as described previously^61^. ErbB2 and ErbB3 staining was conducted by using phycoerythrin-conjugated anti-human ERBB2 (Fab1129P) and ERBB3 (Fab3481P) (R&D Systems)^49^. For flow cytometry analysis, we used BD LSRFortessa X-20 (BD Biosciences) and FlowJo v10. analytical software (Tree Star Inc., Ashland, OR). The dependent measure was mean fluorescent intensity (MFI), providing information on receptor density on the cell membrane.

### Stimulation of TLRs and ErbB

We isolated peripheral blood mononuclear cells and seeded at 1.5 × 10^5^ cells/well into cell plates. After 2 hours, we washed out the non-adherent cells. Adherent monocytes were stimulated for 20 hours with TLR ligands: Gram negative bacterial lipopolysaccharides (LPS) (0.5 lg/mL; TLR4) and flagellin from Salmonella typhimurium (2.5 lg/mL; TLR5) (InvivoGen, San Diego, CA, USA)^47,64^. ErbB receptors were stimulated with NRG1a (296-HR, 65 amino-acid residue recombinant protein from the epidermal growth factor domain of NRG1a (177–241); 200 ng/ml) (R&D system Inc., Minneapolis, MN, USA)^23,25,26^. Following stimulation, we measured the pro-inflammatory cytokines IL-1β, IL-6, and TNF-α, and the anti-inflammatory cytokine IL-10 in the supernatant of monocytes by using a cytometric bead array (CBA) Human Inflammation kit (BD Biosciences) and FACSCanto II (BD Biosciences)^64^.

### Data analysis

STATISTICA 13.0 (Dell Inc.) software was applied for data analysis. After Kolmogorov-Smirnov tests for normal distribution and Levene’s test for homogeneity of variance, analyses of variance (ANOVAs) were used, followed by Tukey Honestly Significant Difference (HSD) tests. Effect size values (η^2^) were calculated in the ANOVAs. The level of statistical significance was set at α < 0.05.
