# Erratum: Uniting the neurodevelopmental and immunological hypotheses: Neuregulin 1 receptor ErbB and Toll-like receptor activation in first-episode schizophrenia

**DOI:** 10.1038/s41598-017-09610-6

**Published:** 2017-08-24

**Authors:** Szabolcs Kéri, Csilla Szabó, Oguz Kelemen

**Affiliations:** 1 Nyírő Gyula Hospital – National Institute of Psychiatry and Addictions, Budapest, Hungary; 2 Department of Cognitive Science, Budapest University of Technology and Economics, Budapest, Hungary; 3 Department of Physiology, Faculty of Medicine, University of Szeged, Szeged, Hungary; 4 Department of Behavioral Sciences, Faculty of Medicine, University of Szeged, Szeged, Hungary

*Scientific Reports* **7**:4147; doi:10.1038/s41598-017-03736-3; Article published online 23 June 2017

The original version of this Article contained an error in the title of the paper, where the word “neurodevelopmental” was incorrectly given as “neuro developmental”. This has now been corrected in the PDF and HTML versions of the Article.

